# Socioeconomic position across the lifecourse & allostatic load: data from the West of Scotland Twenty-07 cohort study

**DOI:** 10.1186/1471-2458-14-184

**Published:** 2014-02-20

**Authors:** Tony Robertson, Frank Popham, Michaela Benzeval

**Affiliations:** 1 Scottish Collaboration for Public Health Research & Policy, University of Edinburgh, 20 West Richmond Street, Edinburgh EH8 9DX, UK; 2 MRC/CSO Social and Public Health Sciences Unit, University of Glasgow, 4 Lilybank Gardens, Glasgow G12 8RZ, UK; 3 Institute for Social & Economic Research, University of Essex, Wivenhoe Park, Essex, Colchester CO4 3SQ, UK

**Keywords:** Epidemiology, Health inequalities, Physiology, Social and Lifecourse Epidemiology

## Abstract

**Background:**

We examined how socioeconomic position (SEP) across the lifecourse (three critical periods, social mobility and accumulated over time) is associated with allostatic load (a measure of cumulative physiological burden).

**Methods:**

Data are from the West of Scotland Twenty-07 Study, with respondents aged 35 (n = 740), 55 (n = 817) and 75 (n = 483). SEP measures representing childhood, the transition to adulthood and adulthood SEP were used. Allostatic load was produced by summing nine binary biomarker scores (1 = in the highest-risk quartile). Linear regressions were used for each of the lifecourse models; with model fits compared using partial F-tests.

**Results:**

For those aged 35 and 55, higher SEP was associated with lower allostatic load (no association in the 75-year-olds). The accumulation model (more time spent with higher SEP) had the best model fit in those aged 35 (b = −0.50, 95%CI = −0.68, −0.32, P = 0.002) and 55 (b = −0.31, 95%CI = −0.49, −0.12, P < 0.001). However, the relative contributions of each life-stage differed, with adulthood SEP less strongly associated with allostatic load.

**Conclusions:**

Long-term, accumulated higher SEP has been shown to be associated with lower allostatic load (less physiological burden). However, the transition to adulthood may represent a particularly sensitive period for SEP to impact on allostatic load.

## Background

Low socioeconomic position (SEP) is associated with greater risk of negative exposures over the lifecourse and has been shown to influence a range of health outcomes, including almost all known morbidities as well as mortality [1-3]. Given the wide range of conditions that vary by SEP, it has been proposed that there are some common biological pathways in how SEP can ‘get under the skin’ [4-7]. Through the exposure to detrimental behavioural, material and psychosocial factors that low SEP results in, the body is put under demands that it can adapt to in the short-term. Allostasis is an active process where, given these exposures, the body attempts to maintain normal system regulation by altering the operating set points or range (‘moving the goalposts’) of the physiological systems involved in adapting and reacting to these conditions. However, if these exposures persist, impairment of the normal regulatory mechanisms can occur (referred to as dysregulation). The cumulative physiological burden on the body that occurs over long spells of such dysregulation is known as allostatic load and is typically irreversible, eventually increasing the risks of poor health and functioning [6,7].

The most widely used construct of allostatic load has been developed by Seeman and colleagues, who have conceptualised it using biomarker measures across an array of systems including the cardiovascular, metabolic and inflammatory systems [7]. This summary measure of allostatic load has been shown to predict the risk of major health outcomes including heart disease and all-cause mortality [7-11]. Importantly, not all of the individual components of allostatic load are risk predictors for these same health outcomes. Assessing these biomarkers together as allostatic load helps us to understand the synergistic nature of the physiological burden on the body imposed by exposure to damaging environmental stressors. This could make allostatic load an important, early predictor of disease risk.

The lifecourse approach in epidemiology understands that health is influenced by social and biological exposures throughout life, while recognising that not all of these exposures (and responses) remain constant over time [12]. To date, there has been consistent (albeit small in number) evidence for lower SEP at specific points in the lifecourse being associated with higher allostatic load, [11,13-15] although how SEP measured across the lifecourse is associated with allostatic load is less well understood. There are three lifecourse models of the actions of SEP on health, known as the accumulation, critical period and mobility models [16]. The accumulation of risk model proposes that long-term exposure to lower SEP results in a proportionate increase in physiological damage and later ill health. The critical/sensitive period model posits that certain points in the lifecourse (especially in utero/early life) can have long-lasting effects on physiological functioning and health later in life [17]. Finally the social mobility model posits that upward social mobility will be beneficial to physiological functioning and health compared to downward mobility or stable SEP over the same time-period. The few studies to incorporate lifecourse measures in the assessment of SEP-allostatic load associations have found mixed results in the associations across the different models, [5,18] but none of them have formally compared the different lifecourse models. The aim of this study was to directly compare the lifecourse models using measures of SEP from three life-stages, modelled against allostatic load. Given the accumulated nature of allostatic load as a measure of physiological burden over time, we hypothesised that lower SEP at each time point in the lifecourse would be associated with higher allostatic load, but that the accumulation of risk model would have the best fit.

## Methods

### Study sample

Data were from the West of Scotland Twenty-07 Study, a community-based, prospective cohort study, which has followed three cohorts of men and women from recruited at the (approximate) ages of 15 (‘1970s cohort’), 35 (‘1950s cohort’) and 55 years (‘1930s cohort’) in 1987 (wave 1/W1) and followed up in a further four waves over the next 20 years. The Study has two subsamples: the regional sample, a two-stage stratified random sample of people living in the Central Clydeside Conurbation, West of Scotland (a socially mixed and mainly urban area) and the localities sample of people from two areas of the city of Glasgow. The target sample at W1 for each cohort was 1,500; the overall achieved sample was 4,510 (1970s n = 1515; 1950s n = 1444; 1930s n = 1551). Baseline respondents have been shown to be representative of the general population of the sampled area [19]. The study design is described in more detail elsewhere [20]. The Tayside Committee on Medical Research Ethics approved the study. Data, including blood samples at wave 5 (W5) (2007/8), were collected by trained nurses in the homes of the study participants when respondents were aged approximately 35 (1970s cohort), 55 (1950s cohort) and 75 (1930s cohort). In 2007/8 2580 took part in W5 (1970s n = 923; 1950s n = 994; 1930s n = 663) and data from 2040 were available for this analysis (1970s n = 740; 1950s n = 817; 1930s n = 483). Approximately 53% of the respondents were female across the cohorts and this was stable over time (Additional file 1: Table S1) [21]. Analysis of baseline data for those who participated at each wave showed that men, people from manual classes (lower SEP) and those with poor starting health were less likely to remain in the study, and in each case this was particularly true of the 1930s cohort. The latter was mainly due to mortality, with nearly 37% of this cohort having died by W5. Among those in the 1950s and 1930s cohorts the proportions reporting poor health increased over time as they aged, but for the 1970s cohort it was relatively stable until the most recent wave when there was a drop in those reporting poor health.

### Biomarkers and allostatic load

Allostatic load was operationalised based on methods described by Seeman et al. [22] and Bird et al. [23]. The biomarkers used represent three physiological systems: cardiovascular [systolic and diastolic blood pressure, and pulse rate]; metabolic [glycated haemoglobin (HbA_1c_), total cholesterol, high density lipoprotein (HDL) cholesterol and waist-hip ratio (WHR)]; and inflammatory [C-reactive protein (CRP) and serum albumin]. In order to account for the effects of medications on biomarker levels, and to reduce the complexity of the final models, respondents’ biomarker values were adjusted as follow. For those on anti-hypertensive medication, systolic and diastolic blood pressures were adjusted by adding 10mmHG and 5mmHG, respectively [24]. Respondents taking diabetes medication had 1% added to their HbA_1c_ values [25]. Where respondents were taking statins, total cholesterol values had 21.24 mg/dL (1.18 mmol/l) added [26]. Where respondents were taking diuretic medication, total cholesterol values were reduced by 4% [27] HDL values were increased by 10% where respondents were taking beta-blockers [27].

Allostatic load was constructed by first dichotomising, separately for each cohort and sex, each of the nine biomarkers based on respondents being in the highest quartile of risk (‘1’) versus the rest (‘0’). These binary measures were then summed to create the overall allostatic load score (ranging from 0 to 9) [23,28].

### Socioeconomic position

SEP was measured at life-stages representing childhood, the transition to adulthood and adulthood. In each case high SEP was coded as ‘1’ and lower SEP as ‘0’. For childhood SEP (SEP1), head of household occupational (parental) social class at age 15 (Registrar General’s 1980 Social Class [29]) was used, asked at wave 1 for all cohorts. For the youngest cohort, the respondents’ parents themselves were asked about their occupations (as respondents were aged 15 at the time), while for the 1950s and 1930s cohort, the question was asked of respondents retrospectively. The six-category variable generated was dichotomised into manual SEP and non-manual SEP. The transitional period from childhood to adulthood (SEP2) was represented by highest educational attainment by W5, dichotomised into having ‘no’ versus ‘some’ formal qualifications. For adult SEP (SEP3), head of household social class was used (as above), based on the most frequently occurring social class for each wave the respondent was of working age and in the study. Where there were equal occurrences of manual and non-manual occupations, the non-manual grouping was selected. Where respondents or their spouses were not in employment at any wave, the respondent’s previous known social class was used.

### Statistical analysis

**Figure 1:**
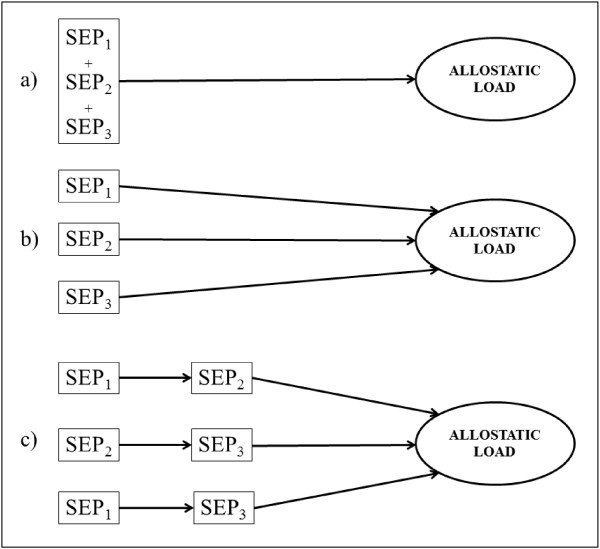
Graphical representation of the different lifecourse models, including the accumulation model (a), the critical periods model (b) and the social mobility model (c).

A structured modelling approach developed by Mishra et al. [30] was used to compare the three theoretical lifecourse models of accumulation (Figure 1a), critical periods (Figure 1b) and social mobility (Figure 1c). The basic idea of this approach is that, given three binary SEP variables, a saturated model would allow all eight possible SEP trajectories to have a different mean outcome. The saturated model is then modelled with three main effects, all two-way interactions, and the 3-way interaction, where the constant (α) is the expected mean for allostatic load for the trajectory where persons were non-manual (higher SEP) at all three time points. This modelling technique allows the direct comparison of each of the different lifecourse models (in the form of the simpler nested models) — accumulation, critical period and mobility hypotheses—to the all-inclusive (saturated) model. Using model-fit statistics, we can identify which of these simpler models has a fit as good as the saturated model. Given its simpler structure, any model found to fit the data as well as the saturated model is considered to be the most parsimonious. This structured modelling approach can provide a formal and clearer understanding of the relative merits of these alternative hypotheses. Table 1 summarises the different lifecourse models. Two versions of the accumulation model were considered. The ‘strict’ model assumes that the longer a person spends in a lower SEP, the worse the physiological burden, irrespective of time period (i.e. having low SEP in childhood and the transition to adulthood will have an identical effect on allostatic load as having low SEP in childhood and adulthood). This model is estimated by constraining the regression coefficient between each SEP measure and allostatic load to an equal value (i.e. the mean effect of the three SEP measures). For the ‘relaxed’ model, each SEP measure is assumed to be contributing to the risk of higher allostatic load, but not necessarily in an equal manner (i.e. there is no such constraint imposed). For the critical period models, a specific SEP life-stage (childhood, the transition to adulthood or adulthood) is considered to only have a relationship with allostatic load, irrespective of other life-stages. This is estimated in the models by constraining the other two of the three SEP measures to equal zero. This is repeated for each of the three life-stages in turn. Finally, we have considered two mobility models. Firstly, early mobility between childhood and the transition to adulthood was considered and secondly mobility between the transition to adulthood and adulthood SEP. To estimate these effects in the models, all other SEP combinations (i.e. low SEP at both life-stages or higher SEP at both life-stages) are constrained to be zero. Only upward and downward mobility are considered, with the assumption that upward mobility will be associated with lower allostatic load and downward mobility with higher allostatic load compared to those showing stable SEP, such that those who remain in a manual social class at both life-stages have equal expected means to those who remain in a non-manual social class at both time points (equal to the constant in the regression model). Full model specifications have been adapted from Mishra et al. [30] and Murray et al. [31] and are available in Additional file 2: Table S2.

**Table 1:** Definitions for each of the analysed Theoretical Lifecourse Models

**Lifecourse model**	**Definition**
**Saturated**	**Combination of all the below models. Given three temporal SEP measures, this allows eight possible SEP trajectories. This is modelled using the main effects from each SEP life-stage, all two-way interactions and the three-way interaction.**
**1a) Accumulation**	**Longer-term exposure to lower SEP results in a proportionate increase in allostatic load.**
- Strict	Regardless of life-stage, the more occasions a respondent spends in a lower SEP environment the greater the effect on raising allostatic load. This is modelled by constraining the effect size between SEP and allostatic load to be equal across all three life-stages.
- Relaxed	SEP at each time-point contributes to the risk of increasing allostatic load, but that these effects do not have to be equal (i.e. can have differing effect sizes in the association with allostatic load).
**1b) Critical**	**Assumes that SEP at only a specific life-stage (childhood, the transition to adulthood or adulthood) will be associated with allostatic load, irrespective of other life-stages. These effects can be modelled by analysing each of the SEP measures of interest (childhood, the transition to adulthood or adulthood SEP) in turn, while constraining the other two life-stages to be zero.**
**1c) Mobility**	**Downward mobility has a negative impact on allostatic load (and the opposite effect for upward mobility).**
- Early	Mobility from childhood to the transitional period between childhood and adulthood (inter-generational mobility).
- Adult	Mobility from the transitional period between childhood and adulthood, to adulthood (intra-generational mobility).*
**No effect**	**Assumes that SEP has no association with allostatic load. Modelled by removing all SEP terms from the regression models.**

*Mobility between childhood and adulthood was not modelled in the analysis.

All models were linear regressions, given the assumption that the nominal allostatic load score is essentially continuous, and adjusted for clustered sampling at baseline using Stata 11. Models were estimated separately for each cohort to assess if SEP was differently associated across the three age groups. Sex was adjusted for in all the models. After removing item-missing data, the complete-case analysis sample sizes were 740, 817 and 483 for the 1970s, 1950s and 1930s cohorts, respectively. All analyses were weighted to the living baseline sample at the time of the wave 5 interviews using inverse probability weights [32]. Weighting the analysis sample in this way inflates the weight for subjects who are underrepresented due to missing data in order to reduce bias introduced by changes in the sample characteristics over time (e.g. those with lower SEP being more likely to drop out of the study). A negative regression coefficient represents lower allostatic load and thus better physiological functioning.

Model-fit was tested by comparing the nested models to the saturated model using a partial F-test. A non-significant *P*-value (*P* > 0.05) indicates that the nested (simpler) model performed as well as the saturated model, following the strategy applied by Mishra et al. [30]. When more than one model fitted the data as well as the saturated model, the model with the highest *P*-value was selected. Where the nested models performed worse than the saturated model (*P* < 0.05), or where the no effect model had a fit no worse than the saturated model (*P* > 0.05), it was concluded that none of the SEP models fitted the data sufficiently. Where all models, including the no effect model, performed worse than the saturated model (*P* < 0.05) it was concluded that the saturated model had the best fit of the data.

## Results

Mean allostatic load ranged from 2.2-2.4 for the three cohorts (Table 2). There was no difference in allostatic load between the sexes. Table 2 also includes the proportions of respondents according to each of the eight possible SEP trajectories. Those respondents with higher SEP at all three life-stages, or higher SEP during both the transition to adulthood and in adulthood, formed the majority of the 1970s (81%) and 1950s cohorts (67%). In comparison, the 1930s cohort had much lower levels of higher SEP, with only 17% having higher SEP at all three life-stages. In the 1970s and 1950s cohorts, those who had higher SEP at all three life-stages had the lowest mean allostatic load. In the 1930s cohort, low allostatic load scores were distributed across several SEP trajectories (Table 2). The highest allostatic load was seen in those who showed higher childhood SEP followed by lower SEP in the remaining life-stages in both the 1970s and 1950s cohorts. In the 1930s cohort, the highest allostatic load scores were in those respondents who had experienced downward, but also upward, mobility in adulthood.

**Table 2:** Proportions of Respondents in each Cohort (1970s, 1950s and 1930s) in each of the eight possible SEP Trajectories, and the mean allostatic load scores (Standard Error)

			**Cohort**					
**SEP**			**1970s**		**1950s**		**1930s**	
**SEP**_**1**_	**SEP**_**2**_	**SEP**_**3**_	**N (%)**	**Mean allostatic load (SE)**	**N (%)**	**Mean allostatic load (SE)**	**N (%)**	**Mean allostatic load (SE)**
0	0	0	13 (2%)	3.5 (0.5)	87 (11%)	2.7 (0.2)	114 (24%)	2.3 (0.2)
1	0	0	3 (1%)	4.3 (2.1)	12 (1%)	3.5 (1.0)	10 (2%)	2.2 (0.4)
0	1	0	72 (9%)	2.9 (0.2)	81 (10%)	2.4 (1.9)	30 (6%)	2.2 (0.3)
0	0	1	13 (2%)	3.3 (0.7)	61 (7%)	2.8 (0.3)	85 (18%)	2.6 (0.5)
1	1	0	28 (4%)	2.2 (0.3)	16 (2%)	2.8 (0.5)	8 (2%)	2.7 (0.7)
1	0	1	4 (1%)	2.6 (0.6)	13 (2%)	2.2 (0.3)	21 (4%)	2.2 (0.3)
0	1	1	306 (41%)	2.5 (0.2)	312 (38%)	2.1 (0.1)	132 (27%)	2.3 (0.1)
1	1	1	301 (40%)	2.0 (0.1)	235 (29%)	1.9 (0.1)	83 (17%)	2.2 (0.2)
			740 (100%)	2.4 (0.1)	817 (100%)	2.3 (0.1)	483 (100%)	2.3 (0.1)

Where, SEP_1_ = childhood SEP; SEP_2_ = SEP during the transition from childhood to adulthood; SEP_3_ = adulthood SEP.

In the 1970s cohort, higher SEP was associated with lower allostatic load at all three critical periods and accumulated over time (Table 3). Upward mobility between childhood and the transition to adulthood was associated with higher allostatic load (in contrast to the expected direction); although none of the other mobility measures were statistically significant. However, it was the relaxed accumulation model that had the best fit of all the models (P = 0.93). A longer time spent in a higher SEP was associated with lower allostatic load, but the association was only significant in childhood for the measure (b = −0.51, 95% CI = −0.90, −0.13, P = 0.01) and the transition to adulthood (b = −0.84, 95% CI = −1.63, −0.05, P = 0.04), but not in adulthood (b = −0.33, 95% CI = −0.83, 0.17, P = 0.19).

**Table 3:** Parameter estimates and P-values for the model fits of each of the theoretical lifecourse socioeconomic models tested against a saturated model

	**1970s cohort**			**1950s cohort**			**1930s cohort**		
**SEP model**	**P for model fit**	**b**	**CI (95%)**	**P for model fit**	**b**	**CI (95%)**	**P for model fit**	**b**	**CI (95%)**
**1a) Accumulation**									
- Strict	0.90	−0.50	−0.68, −0.32**	**0.54**	**−0.31**	−0.49, −0.12**	0.99	−0.01	−0.17, 0.16
**- Relaxed**	**0.93**			0.40			0.99		
**SEP**_**1**_		**−0.51**	−0.90, −0.13**		−0.15	−0.45, 0.15		−0.10	−0.50, 0.30
**SEP**_**2**_		**−0.84**	−1.63, −0.05*		−0.52	−0.97, −0.06*		−0.10	−0.77, 0.57
**SEP**_**3**_		**−0.33**	**−0.83, 0.17**		−0.24	−0.62, 0.13		0.16	−0.58, 0.91
**1b) Critical period**									
- Childhood	0.30	−0.59	−0.94, −0.24**	0.02	−0.33	−0.64, −0.01*	0.99	−0.09	−0.54, 0.36
- Early adulthood	<0.01	−1.04	−1.85, −0.23*	0.12	−0.65	−1.09, −0.22**	0.99	−0.54	−1.62, 0.53
- Adulthood	0.02	−0.57	−1.03, −1.11*	0.13	−0.47	−0.85, −0.10*	0.99	0.10	−0.39, 0.59
**1c) Social mobility**									
- Early	0.01			0.07			0.97		
Upward		0.44	0.09, 0.80*		−0.09	−0.35, 0.18		−0.03	−0.48, 0.41
Downward		1.09	−0.95, 3.13		0.67	−0.55, 1.89		−0.14	−0.73, 0.45
- Adult	<0.01			0.08			0.97		
Upward		0.68	−0.47, 1.83		0.57	0.05, 1.09*		0.26	−0.67, 1.19
Downward		0.36	−0.19, 0.91		0.30	−0.05, 0.66		0.13	−0.48, 0.74
**No effect**	<0.01			0.02			**1.00**		

Where, SEP_1_ = childhood SEP; SEP_2_ = SEP during the transition from childhood to adulthood; SEP_3_ = adulthood SEP.

Bold = best model fit.

*P ≤ 0.05.

**P ≤ 0.01.

For the 1950s cohort, higher SEP at each of the three life-stages was significantly associated with lower allostatic load, as was accumulated higher SEP over time. SEP mobility was not associated with allostatic load. However, it was the strict accumulation model that had the best model fit (P = 0.54), with more occasions spent in a higher SEP associated with lower allostatic load (b = −0.31, 95% CI = −0.49, −0.12, P = 0.002) (Table 3). Although all three life-stages were associated with allostatic load in the critical period models, the relaxed accumulation model showed that each of the three time-points had associations with allostatic load that varied in strength. However, these differences were not sufficient to improve the fit of the model over the saturated one. The transition to adulthood was the only SEP measure to remain significant when all three measures were simultaneously modelled in the relaxed accumulation model (b = −0.52, 95% CI = −0.97, −0.06, P = 0.03).

Unlike the 1970s and 1950s cohorts, the no effect model had the best model fit in the 1930s cohort (Table 3), indicating there was no association between SEP and allostatic load. This reflected lack of variation in the mean allostatic load scores by the eight SEP trajectories (Table 2).

## Discussion

This study has found evidence for higher SEP across the lifecourse to be associated with lower allostatic load (better physiological functioning) in respondents under age 75 from the West of Scotland. Comparing different theoretical lifecourse models of SEP revealed that accumulated SEP across the lifecourse had the best model fit to explain the data in those aged 35 and 55, but there was no association between SEP and allostatic load in those aged 75.

### Findings in relation to other studies

Although a small number of studies have tested for associations between SEP and allostatic load, there has been limited attention given to differences in the association across the lifecourse and no studies have compared the different lifecourse models directly using a structured modelling approach. Gruenewald et al. [5] found that higher allostatic load was associated with lower SEP accumulated across the lifecourse, at critical periods (childhood and adulthood) and a gradient according to social mobility. However, there was no formal comparison between the lifecourse models. In two studies of Swedish men and women, accumulated low SEP (over four time-points) was associated with the highest allostatic load scores [18,33]. Critical period models reflected a mixture of null and negative associations (lower SEP and higher allostatic load). These results largely match with the overall social patterning seen in allostatic load in this study, particularly with accumulated SEP, although studies on SEP and allostatic load remain limited to a few cohorts, mainly with low sample sizes.

### Strengths and limitations

This study has built on the findings of other studies with regards allostatic load and SEP, as well as focusing on the lifecourse nature of SEP and health/physiological burden and directly comparing the different lifecourse models using a structured modelling approach. Furthermore, this study has also included both genders and three age cohorts representing individuals in early-, mid- and late-adulthood in a relatively large study. However, there remain some limitations.

We have followed a modelling approach which uses binary SEP measures at different stages of the lifecourse [30]. Reducing these indicators to binary variables does simplify the information measured and may not allow us to identify non-linear patterns of association with allostatic load. Using SEP measures with multiple categories would increase the complexity of the models, as well as increasing less common SEP trajectories. Another potential limitation is the age structure of the Twenty-07 Study, made up of the three cohorts, each 20 years apart. This lack of a continuous age range limits the conclusions that can be made about the ages not sampled here, although it gives a good indication of the association at key life-stages. Our allostatic load construct did not contain any markers from the hypothalamic pituitary adrenal (HPA) axis that forms part of the neuroendocrine system (stress response). The stress response is believed to play a key role in allostasis and subsequent allostatic load, with a cascade of events that starts with primary stress mediators, such as cortisol, before initial stress responses (‘primary effects’ such as rapid increases in blood pressure and sugars/fats that supply the body with extra energy) and then to secondary and tertiary outcomes (measured in our allostatic load model). These stress markers are difficult to measure in large surveys where direct examination of the stress response (e.g. measuring cortisol) is difficult due to the circadian rhythms shown in these stress hormones and the rapid sampling required in order to measure baseline versus activated levels. Inclusion of measures such as cortisol could improve the power of allostatic load as an earlier risk predictor for disease, but their exclusion does not invalidate this allostatic load construct as the subsequent outcomes of cortisol release are still being included.

### Meaning of findings

Given that the concept of allostatic load is defined as the “long-term accumulation and gradual development of physiological dysregulation”, it is conceptually well matched to the lifecourse approach to understanding the effects of SEP on health [5,18]. The accumulation model represents long-term exposure to potentially damaging environmental exposures (e.g. pollution, carcinogens or violent crime), psychosocial exposures (e.g. stress or lack of control) and health-damaging behaviours (e.g. smoking, consuming alcohol or having a poor diet), factors that would be expected to cause the most damage to a person’s physiological systems given longer-term exposure. Although the accumulation model was identified as the best-fit model in the 1970s and 1950s cohorts (with a longer time spent with higher SEP associated with lower allostatic load), it was apparent, given the relaxed accumulation model was the best-fit model in the 1970s cohort and there were significant life-stage differences in the 1950s cohort, that a simple ‘summing of the risk’ approach (assuming all life-stages pose identical risks) might not allow us to fully understand the changing nature of the association between allostatic load and SEP over the lifecourse. For the 1970s cohort, childhood and the transition to adulthood appear to be particularly sensitive periods for the association between SEP and allostatic load, while for the 1950s cohort it was the transition to adulthood. At different stages in the lifecourse, SEP can represent different exposures to risk or protection. Childhood SEP, in this case the head of household occupation/social class of a respondent’s parents, might represent both material and psychosocial factors important for health at that time, as well as influencing the opportunities (e.g. jobs, education etc.), exposures (e.g. neighbourhood conditions) and coping skills (e.g. IQ, self-esteem) that are important factors also in later life. The transitional period between childhood and adulthood, measured here as educational attainment, can represent similar provision of skills and opportunities for later life, but may also represent a sensitive period in the transition from childhood to adulthood, where psychosocial, physical and behavioural factors can all play an important role in influencing health [17].

In two of the three cohorts higher accumulated SEP was associated with lower allostatic load scores, but this association was not seen in the 1930s cohort. One issue in studies of older individuals is the potential for survival bias. Greater numbers from the 1930s cohort had been lost to the study compared to the 1950s and 1970s cohorts, reducing the 1930s sample size and subsequent statistical power, with the risk of death higher in those individuals with lower SEP and poorer health (Additional file 3: Table S3). These individuals potentially have greater levels of physiological damage and higher allostatic load, thereby reducing the observed associations at older ages. Correcting the analyses using weights addresses some of the issues of selective dropout (higher in lower SEP individuals), but does not correct for survival bias. Alternative to survival bias, there is the possibility for age and cohort effects. Age effects are where the relationship between SEP and allostatic load changes as people age. The SEP-allostatic load association seen in younger individuals may become diluted as other factors such as disease have a greater influence on health and physiological functioning as people age. Cohort effects are also possible, with other studies having found that the association between SEP and health is stronger in younger birth cohorts [34]. It has been suggested that this is the result of the changing contexts for the SEP-health association. For example, the meaning of SEP has changed for the different cohorts (e.g. the growing importance of education in people’s lives with younger birth cohorts); life expectancy has increased with younger cohorts (i.e. they may be physiological ‘younger’ at older ages than previous cohorts); and the pattern of diseases has also altered across cohorts (e.g. shift from higher prevalence of communicable to non-communicable disease, with these different disease-types potentially impacting differently on physiological burden across the body).

## Conclusions

Allostatic load is a concept that aims to measure the cumulative physiological burden on the body that occurs across multiple physiological systems by measuring, and combining, multiple biomarkers. Although a small number of studies have tested for associations between SEP and allostatic load, there has been limited attention given to differences in the association across the lifecourse and no studies have compared the different lifecourse models directly using a structured modelling approach. This study is the first to statistically compare the different SEP lifecourse models for their association with allostatic load. Using this lifecourse approach, we identified that accumulated SEP was the strongest predictor of allostatic load, although a simple ‘summing of the risk’ may not encapsulate the entire nature of the association between allostatic load and SEP across the lifecourse. This finding highlights that when considering the links between socioeconomic circumstances and allostatic load (and health more generally), we must consider the association across the lifecourse and not assume short-term interventions will have significant effects.

## Competing interests

The authors have no competing interests to report.

## Authors’ contributions

TR conceived of the study, contributed to the design of the study, conducted the analysis and wrote the draft manuscripts. FP and MB contributed to the design and analysis strategy of the study and commented on manuscript drafts. All authors read and approved the final manuscript.

## Pre-publication history

The pre-publication history for this paper can be accessed here:

http://www.biomedcentral.com/1471-2458/14/184/prepub

## Supplementary Material

Additional file 1: Table S1 Sociodemographic information for respondents from the Twenty-07 Study, Waves 1 – 5 (Adapted from Benzeval et al. [21]). Click here for file

Additional file 2: Table S2 Model specifications (Adapted from Mishra et al. [30] and Murray et al. [31]). Click here for file

Additional file 3: TableS3 Odds ratios for the Odds of Death by Wave 5 given Socioeconomic and Health Characteristics at Wave 1. Click here for file
