# Supplementary material for: Socioeconomic position across the lifecourse & allostatic load: data from the West of Scotland Twenty-07 cohort study
Source: BMC Public Health. 2014 Feb 20;14:184. doi: 10.1186/1471-2458-14-184 (PMC3942053; doi:10.1186/1471-2458-14-184)
Supplement: Additional file 1: Table S1 — Sociodemographic information for respondents from the Twenty-07 Study, Waves 1 – 5 (Adapted from Benzeval et al. [21]). [file 1471-2458-14-184-S1.docx]

Additional file 1: Table S1 Sociodemographic information for respondents from the Twenty-07 Study, Waves 1 – 5 (Adapted from Benzeval et al., 2010 [[21](#_ENREF_21)])

| **Characteristics** | **Baseline**  **1987/8** | **Wave2**  **1990/2** | **Wave3**  **1995/7** | **Wave4**  **2000/4** | Wave 5  **2007/8** |
| --- | --- | --- | --- | --- | --- |
| **1970s Cohort** |  |  |  |  |  |
| Number in cohort at each wave (N) | 1515 | 1343 | 916 | 843 | 942 |
| Average Age | 15.7 | 18.6 | 24.8 | 30.2 | 36.7 |
|  |  |  |  |  |  |
| % of whole sample in cohort at each wave | 33.6 | 35.0 | 30.8 | 31.7 | 36.2 |
| % of cohort female | 51.3 | 52.4 | 54.1 | 54.4 | 54.9 |
| % of cohort in poor health at baseline^b^ | - | - | - | - | - |
| % of cohort in poor health status at each wave | - | 33.6 | 30.5 | 32.5 | 22.9 |
| % of cohort in manual class at baseline | 39.5 | 37.1 | 34.5 | 33.7 | 34.7 |
| % of cohort in manual class at each wave | 39.5 | 37.5 | 32.1 | 20.4 | 17.1 |
| % of cohort dead (of baseline sample) | 0 | 0.1 | 0.5 | 1.1 | 1.7 |
| **1950s Cohort** |  |  |  |  |  |
| Number in cohort at each wave (N) | 1444 | 1225 | 1026 | 980 | 999 |
| Average Age | 36.2 | 40.5 | 45.2 | 50.2 | 57.1 |
|  |  |  |  |  |  |
| % of whole sample in cohort at each wave | 32.0 | 32.0 | 34.5 | 36.8 | 38.4 |
| % of cohort female | 54.6 | 55.2 | 55.6 | 54.5 | 54.3 |
| % of cohort in poor health at baseline | 23.8 | 23.8 | 21.9 | 21.6 | 21.8 |
| % of cohort in poor health status at each wave | 23.8 | 28.8 | 31.2 | 33.1 | 29.5 |
| % of cohort in manual class at baseline | 34.2 | 33.5 | 31.1 | 29.8 | 30.4 |
| % of cohort in manual class at each wave | 34.2 | 30.5 | 27.7 | 26.9 | 26.3 |
| % of cohort dead (of baseline sample) | 0 | 0.6 | 2.1 | 3.9 | 6.0 |
| **1930s Cohort** |  |  |  |  |  |
| Number in cohort at each wave (N) | 1551 | 1266 | 1030 | 838 | 663 |
| Average Age | 56.2 | 59.6 | 64.4 | 69.1 | 76.2 |
|  |  |  |  |  |  |
| % of whole sample in cohort at each wave | 34.4 | 33.0 | 34.7 | 31.5 | 25.5 |
| % of cohort female | 54.7 | 54.2 | 56.3 | 56.1 | 57.9 |
| % of cohort in poor health at baseline | 41.9 | 41.9 | 39.6 | 36.0 | 33.8 |
| % of cohort in poor health status at each wave | 41.9 | 39.5 | 44.3 | 40.1 | 46.2 |
| % of cohort in manual class at baseline | 45.8 | 43.4 | 39.9 | 37.0 | 34.2 |
| % of cohort in manual class at each wave | 45.8 | 45.8 | 43.3 | 42.4 | 41.2 |
| % of cohort dead (of baseline sample) | 0 | 4.8 | 11.7 | 23.1 | 36.6 |
| **All cohorts** |  |  |  |  |  |
| Number in cohort at each wave (N) | 4510 | 3834 | 2972 | 2661 | 2604 |
| Average Age | 36.2 | 39.2 | 45.6 | 49.8 | 54.6 |
|  |  |  |  |  |  |
| % female | 53.5 | 53.9 | 55.4 | 55.0 | 55.4 |
| % poor health at baseline^e^ | 33.2 | 33.0 | 30.8 | 28.3 | 26.6 |
| % poor health status at each wave | 33.2 ^d^ | 34.0 | 35.5 | 35.1 | 31.4 |
| % in manual class at baseline | 40.0 | 38.0 | 35.2 | 33.3 | 32.9 |
| % in manual class at each wave | 40.0 | 38.0 | 34.5 | 29.7 | 26.8 |
| % dead (of baseline sample) | 0 | 1.9 | 4.9 | 9.6 | 15.1 |

^a^Data in this column represent person-waves

^b^The self-assessed health question used in this analysis was not included in the baseline interview for the 1970s cohort

^c^Household class from the previous (or most recent) wave is used for the person-wave data as this is what was used in the statistical models.

^d^This value represent the percentage of person-waves where the respondent is actually dead.

^e^The self-assessed health question used in this analysis was not included in the baseline interview for the 1970s cohort, and so these figures give combined percentages for the 1950s and 1930s cohorts only.
