# Supplementary material for: Socioeconomic position across the lifecourse & allostatic load: data from the West of Scotland Twenty-07 cohort study
Source: BMC Public Health. 2014 Feb 20;14:184. doi: 10.1186/1471-2458-14-184 (PMC3942053; doi:10.1186/1471-2458-14-184)
Supplement: Additional file 2: Table S2 — Model specifications (Adapted from Mishra et al. [30] and Murray et al. [31]). [file 1471-2458-14-184-S2.docx]

Additional file 2: Table S2 Model specifications (Adapted from Mishra et al., 2009 [[30](#_ENREF_30)] and Murray et al., 2011[[31](#_ENREF_34)])

| **Model** | **Lifecourse model specification** | **Constraints** |
| --- | --- | --- |
| **Saturated** | α + β_1_+ β_2_ + β_3_ + β_12_ + β_13_ + β_23_ + β_123_ |  |
| **1a) Accumulation** |  |  |
| - Strict | α + β_1_+ β_2_ + β_3_ | β_1_ = β_2_ = β_3_; β_12_ = β_23_ = β_13_ = β_123_ = 0 |
| - Relaxed | α + β_1_+ β_2_ + β_3_ | β_12_ = β_23_ = β_13_ = β_123_ = 0 |
| **1b) Critical Period** |  |  |
| - Childhood | α + β_1_ | β_2_ = β_3_ = β_12_ = β_23_ = β_13_ = β_123_ = 0 |
| - Transition to Adulthood | α + β_2_ | β _1_ = β_3_ = β_12_ = β_23_ = β_13_ = β_123_ = 0 |
| - Adulthood | α + β_3_ | β _1_ = β_2_ = β_12_ = β_23_ = β_13_ = β_123_ = 0 |
| **1c) Social Mobility** |  |  |
| - Early* | α + β_1_+ β_2_ + β_12_ | β_12_ =-(β_1_+ β_2_); β_3_ = β_23_ = β_13_ = β_123_ = 0 |
| - Adult** | α + β_2_+ β_3_ + β_23_ | β _23_ =-(β_2_+ β_3_); β_1_ = β_12_ = β_13_ = β_123_ = 0 |
| **No Effect** | α | β_1_= β_2_ = β_3_ = β_12_ = β_13_ = β_23_ = β_123_ = 0 |

Where α = constant/intercept; β = regression coefficient / slope; 1 = SEP_1_; 2 = SEP_2_; 3 = SEP_3_

* Early mobility = α + upward mobility + downward mobility

= α + β_1_(1- β_2_) + β_2_(1- β_1_)

= α + β_1_ + β_2_ – (β_1_ + β_2_)

* Adult mobility = α + upward mobility + downward mobility

= α + β_2_(1- β_3_) + β_3_(1- β_2_)

= α + β_2_ + β_3_ – (β_2_ + β_3_)
