# Supplementary material for: Socioeconomic position across the lifecourse & allostatic load: data from the West of Scotland Twenty-07 cohort study
Source: BMC Public Health. 2014 Feb 20;14:184. doi: 10.1186/1471-2458-14-184 (PMC3942053; doi:10.1186/1471-2458-14-184)
Supplement: Additional file 3: TableS3 — Odds ratios for the Odds of Death by Wave 5 given Socioeconomic and Health Characteristics at Wave 1. [file 1471-2458-14-184-S3.docx]

Additional file 3: Table S3 Odds ratios for the Odds of Death by Wave 5 given Socioeconomic and Health Characteristics at Wave 1

|  | **1970s** | | | | **1950s** | | | | **1930s** | | | |
| --- | --- | --- | --- | --- | --- | --- | --- | --- | --- | --- | --- | --- |
|  | **Odds Ratio** | **95% CI (low)** | **95% CI (high)** | ***P*** | **Odds Ratio** | **95% CI (low)** | **95% CI (high)** | ***P*** | **Odds Ratio** | **95% CI (low)** | **95% CI (high)** | ***P*** |
| **Social Class** |  |  |  |  |  |  |  |  |  |  |  |  |
| Non-manual vs. Manual | 0.465 | 0.185 | 1.171 | **0.104** | 0.376 | 0.226 | 0.627 | **<0.001** | 0.643 | 0.518 | 0.797 | **<0.001** |
| **Area deprivation*** |  |  |  |  |  |  |  |  |  |  |  |  |
| Less deprived vs. Most deprived | 0.891 | 0.402 | 1.975 | **0.776** | 0.458 | 0.297 | 0.709 | **<0.001** | 0.670 | 0.543 | 0.826 | **<0.001** |
| **Home ownership** |  |  |  |  |  |  |  |  |  |  |  |  |
| Owner vs. Renter | 0.310 | 0.116 | 0.831 | **0.020** | 0.242 | 0.151 | 0.388 | **<0.001** | 0.592 | 0.479 | 0.733 | **<0.001** |
| **Employment** |  |  |  |  |  |  |  |  |  |  |  |  |
| Employed vs. Not employed | - | - | - | **-** | 0.465 | 0.299 | 0.721 | **0.001** | 0.571 | 0.463 | 0.704 | **0.571** |
| **Limiting Longstanding Illness** |  |  |  |  |  |  |  |  |  |  |  |  |
| No vs. Yes | 1.230 | 0.287 | 5.272 | **0.780** | 0.632 | 0.382 | 1.045 | **0.074** | 0.507 | 0.405 | 0.634 | **<0.001** |
| **Lung function†** |  |  |  |  |  |  |  |  |  |  |  |  |
| High FEV1 vs. Low FEV1 | 0.449 | 0.103 | 1.952 | **0.286** | 0.397 | 0.216 | 0.729 | **0.003** | 0.805 | 0.652 | 0.994 | **0.044** |
| **Blood pressure‡** |  |  |  |  |  |  |  |  |  |  |  |  |
| Low vs. High | 0.903 | 0.120 | 6.795 | **0.921** | 0.572 | 0.355 | 0.923 | **0.022** | 0.823 | 0.663 | 1.022 | **0.078** |
| **Self-rated health** |  |  |  |  |  |  |  |  |  |  |  |  |
| Good/Excellent vs. Poor/Fair | 0.473 | 0.109 | 2.055 | **0.318** | 0.473 | 0.299 | 0.749 | **0.001** | 0.452 | 0.364 | 0.562 | **<0.001** |
| **Sex** |  |  |  |  |  |  |  |  |  |  |  |  |
| Female vs. Male | 0.364 | 0.151 | 0.876 | **0.024** | 0.513 | 0.329 | 0.800 | **0.003** | 0.585 | 0.474 | 0.720 | **<0.001** |
|  |  |  |  |  |  |  |  |  |  |  |  |  |

* Less deprived = Depcat ≤ 5. More deprived = Depcat >5

† FEV1 = Forced expiratory Volume in one second. FEV1 is a measure of lung function where higher values represent better lung functioning. Low FEV1 = worst quintile of FEV1 values

‡ Low blood pressure = worst quintile of diastolic blood pressure values
